# Comparing high-flow nasal cannula and non-invasive ventilation in critical care: insights from deep counterfactual inference

**DOI:** 10.1038/s44401-025-00049-w

**Published:** 2025-12-04

**Authors:** Xiaolei Lu, Michael Miller, Alex K. Pearce, Jonathan Y. Lam, Aaron E. Boussina, Kai Zheng, Atul Malhotra, Shamim Nemati

**Affiliations:** 1 Department of Biomedical Informatics, University of California San Diego, La Jolla, CA USA; 2 Division of Pulmonary, Critical Care, and Sleep Medicine, University of California San Diego, La Jolla, CA USA; 3 Department of Informatics, University of California, Irvine, Irvine, CA USA

**Keywords:** Diseases, Health care, Medical research, Risk factors

## Abstract

Randomized trials comparing high-flow nasal cannula (HFNC) and non-invasive positive pressure ventilation (NIV) for acute respiratory failure (ARF) offer population-level guidance but often fail to capture individual variability in treatment response. In this retrospective study, we identified intensive care units (ICU) patients at risk of invasive mechanical ventilation (IMV) using a previously published risk prediction model. Patients who first received HFNC or NIV after crossing the high-risk threshold formed the early treatment cohort. We developed a deep counterfactual model that integrates representation learning, conditional normalizing flows, and confounder adjustment to estimate individualized treatment effects (ITEs) between HFNC and NIV. Treatment concordance, defined as alignment between the model’s recommendation and the treatment actually administered, was assessed using multivariate logistic regression. At UC San Diego Health (UCSD), concordant treatment was associated with significantly reduced odds of IMV (odds ratio [OR] = 0.661 for NIV; 0.677 for HFNC) and mortality or hospice discharge (OR = 0.679 for NIV; 0.749 for HFNC). At UC Irvine Health (UCI), concordant treatment was also linked to improved outcomes, particularly for mortality or hospice discharge (OR = 0.092 for NIV; 0.088 for HFNC). These findings highlight the value of individualized, model-guided respiratory support strategies in improving outcomes for critically ill patients.

## Introduction

Acute respiratory failure (ARF) is a common reason for hospitalization, both in general wards and intensive care units (ICU). Oxygen therapy, generally with simple nasal cannula, is often the initial treatment modality for those patients with hypoxemia. If respiratory failure progresses, and patients require >6−8 L/min of oxygen to maintain adequate oxygen saturation or exhibit respiratory distress or hypercarbia, treating physicians escalate respiratory support in several different way including high flow nasal cannula (HFNC; airflows up to 50−60 L/min), non-invasive positive pressure ventilation (NIV), or invasive mechanical ventilation (IMV). While the choice of the initial treatment modality is important and could influence patient outcomes, there remains clinical equipoise surrounding this treatment decision as several randomized controlled trials have shown mixed results^1–10^.

Medical society guidelines provide general guidance and advice for some specific clinical contexts. For example, the European Respiratory Society (ERS) and American Thoracic Society (ATS) guidelines^11^ support NIV use for acute exacerbation of chronic obstructive pulmonary disease (AECOPD), cardiogenic pulmonary edema, immunocompromised patients with ARF, post-operative respiratory failure, palliative treatment of dyspnea, chest trauma patients with ARF, and preventing post-extubation respiratory failure in high-risk patients. However, the guidelines do not make recommendations about asthma, de novo ARF, or pandemic viral illness. Guideline updates from the ERS in 2022^12^ added that patients with acute hypoxemia respiratory failure should generally be treated with HFNC over NIV, HFNC or NIV is equivocal for post-operative patients at high-risk of respiratory complications, and removed the recommendation for NIV use in immunocompromised patients. Many of these recommendations are conditional with low certainty of evidence. Real-world clinical care is often complicated, and patients can have multiple co-occurring conditions with varying indications or contraindications (e.g., multifocal pneumonia, pulmonary edema and AECOPD, neuromuscular weakness, facial trauma or surgery, large beards, inability to manage secretions or protect airway, severe encephalopathy, hemodynamic instability, gastric or esophageal pathology, etc.) for either HFNC or NIV.

Another feature complicating decisions regarding oxygen delivery modality is that ICU patients themselves are often inhomogeneous and many of the conditions commonly encountered in the ICU (sepsis, ARF, etc.) are syndromes composed of a variety of pathophysiologic states and severities. Controlling a myriad of disease states and patient-specific differences in randomized clinical trials in this population is difficult. It is therefore unsurprising that trials in this arena commonly fail to demonstrate a difference between treatment and control groups, show mixed results among studies, or fail to provide generalizable results. One explanation for these mixed findings is that the average treatment effect (ATE) captured by randomized control trials (RCTs) may hide variations of the treatment’s effect on a clinical outcome across levels of patient characteristics. Another way to rephrase is that even with a null overall result, due to heterogeneity of treatment effect (HTE), some patients may benefit from an intervention, others are unchanged, and others may even be hurt.

Individualized treatment effect (ITE) predictive modeling provides patient-specific estimates of treatment response to capture heterogeneity in how patients respond to interventions. Within the area of machine learning (ML), counterfactual models, often applied to observational data, have been developed within the potential outcome framework to estimate the outcomes of different treatment strategies by simulating “what-if” scenarios^13–16^. Deep counterfactual models build on this idea by incorporating complex clinical data and adjusting for measured confounding biases, such as patient demographics, comorbidities, prior treatments, and disease progression, to provide more accurate estimates of treatment effects^17,18^. By generating these counterfactual predictions, ML can aid clinicians to identify which therapies are most likely to benefit individual patients. However, most counterfactual models fail to account for unmeasured confounders, including clinical decision-making biases and hidden patient severity factors that influence treatment decisions. Additionally, the process of representation learning in deep counterfactual models can lead to the loss of important measured confounders^19^, which further impacts the ITE estimation.

We conducted a retrospective analysis of ICU patients at risk of progressing to IMV identified using the Vent.io respiratory failure risk prediction model^20^. To estimate the ITE of HFNC versus NIV as the initial respiratory support, we developed a deep counterfactual model RepFlow-CFR, a flow-based confounder adjustment framework that integrates representation learning, normalizing flows and counterfactual inference, which was designed to account for both measured and unmeasured confounders while improving causal effect estimation. We tested the hypothesis that the deep counterfactual model (1) would accurately identify the patients who would benefit from either HFNC or NIV, and (2) concordance between the model’s recommendations and the actual treatment administered is associated with improved clinical outcomes.

## Results

In this section, we first identified high-risk patients using the Vent.io model and defined the Post-T0 initial HFNC/NIV cohorts at two academic ICUs. We then evaluated the predictive performance of our RepFlow-CFR model in forecasting the need for IMV, both before and after site-specific fine-tuning. Next, we analyzed ITE to categorize patients by likely benefit from HFNC or NIV, and assessed differences in clinical characteristics and outcomes across groups. We further examined the top features contributing to ITE predictions using SHAP analysis. Finally, we evaluated whether concordance between model-recommended and actual treatments was associated with improved clinical outcomes, using both descriptive statistics and multivariable regression models across two sites.

### Identification of post-T0 initial HFNC/NIV cohort

UCSD ICU cohort consists of 31,180 encounters, and UCI ICU cohort includes 3290 encounters. Using the pretrained Vent.io, which was developed based on UCSD ICU cohort (Supplementary 5), we identified 5685 encounters for UCSD high-risk cohort and 578 encounters for UCI high-risk cohort. The Vent.io achieved an AUC of 0.895, sensitivity of 0.603, specificity of 0.845 and positive predictive value of 0.177 at UCSD and an AUC of 0.842, sensitivity of 0.547, specificity of 0.836 and positive predictive value of 0.167 at UCI. Patient characteristics for these two ICU cohorts are reported in Table S5. We then filtered and selected patients who meet the criteria of first receiving either HFNC or NIV after Vent.io T0, with 1956 encounters in UCSD Post-T0 initial HFNC cohort and 169 encounters in UCI Post-T0 initial HFNC/NIV cohort. Table 1 shows baseline characteristics in UCSD and UCI Post-T0 initial HFNC/NIV cohorts. NIV-treated group has a higher Charlson Comorbidity Index (CCI) score and a higher prevalence of Chronic Obstructive Pulmonary Disease (COPD) and Congestive Heart Failure (CHF) compared to the HFNC-treated group at both sites.

**Table 1:** Baseline characteristics of patients in UCSD and UCI Post-T0 initial HFNC/NIV cohorts

Variable	UCSD (development site)		UCI (validation site)	
	NIV-treated*	HFNC-treated*	NIV-treated*	HFNC-treated*
**Characteristic**				
Encounters, N	591	1365	38	131
Age(years), mean (SD)	63(16.3)	61(16.6)	66(15.0)	63(17.6)
**Gender, N (%)**				
Male	369(62.4)	806(59.0)	20(52.6)	86(65.6)
**Organ dysfunction, median (IQR)**				
**Charlson Comorbidity Index, Median (IQR)**	3.0(1.0-5.0)	2.0(1.0-5.0)	3.0(1.0–6.0)	2.0(0.0-3.0)
Congestive Heart Failure Component, N (%)	245(41.5)	315(23.1)	15(39.5%)	29(22.1)
Chronic Obstructive Pulmonary Disease, N (%)	161(27.2)	234(17.1)	4(10.5%)	8(6.1)
SOFA^a^ score (at Vent.io T0), Median (IQR)	1.0(0.0-3.0)	1.0(0.0-3.0)	2.0(1.0-4.0)	1.0(0.0-3.0)
**Outcomes, N (%)**				
IMV^b^	122 (20.6)	334 (24.5)	9 (23.7)	38 (29.0)
Mortality	147 (24.9)	431(31.6)	9 (23.7)	29 (22.1)
Hospice	6 (1.0)	11(0.8)	5 (13.2)	31 (23.7)

*The HFNC-treated and NIV-treated refer to the actual initial treatments administered to patients after the Vent.io T0 timepoint.

^a^ SOFA: Sequential Organ Failure Assessment.

^b^ We counted the number of patients for each initial ventilation including both its individual use and any combined usage methods categorized under the same category.

### IMV predictive performance

RepFlow-CFR achieved an AUC of 0.820 and a PR-AUC of 0.566 on the UCSD Post-T0 initial HFNC cohort, which is comparable to the baseline CFR model (AUC: 0.821, PR-AUC: 0.571). However, when externally validated on the UCI Post-T0 initial HFNC/NIV cohort, performance declined, with RepFlow-CFR achieving an AUC of 0.630 and a PR-AUC of 0.444, compared to CFR’s AUC of 0.656 and PR-AUC of 0.415. To improve generalizability, we fine-tuned both CFR and RepFlow-CFR using a random 25% of the UCI Post-T0 initial HFNC/NIV cohort. After fine-tuning, RepFlow-CFR achieved an AUC of 0.727 and a PR-AUC of 0.553, while CFR achieved an AUC of 0.758 and a PR-AUC of 0.590 on the UCI site.

### Predicted individualized treatment effects

To categorize patients based on their predicted ITE, we defined absolute ITE ≤ 0.001 as indicating patients who are non-responders or indifferent to both treatments despite minor fluctuations in ITE estimates, and divided the patients into three groups: NIV preferred (ITE < -0.001), HFNC preferred (ITE > 0.001), or Indifferent (ITE between -0.001 and 0.001). This band buffers near-zero estimation noise and discourages acting on ≤0.1-percentage-point risk differences. Table 2 summarizes the characteristics of patients by the predicted ITE of RepFlow-CFR model. At UCSD, the HFNC preferred group exhibited the highest CCI and showed a greater prevalence of CHF and COPD compared to the NIV preferred group. Rates of IMV were highest in the NIV preferred group at both UCSD (24.9%) and UCI (37.9%). Meanwhile, mortality and hospice outcomes were not significantly different across treatment groups at either site. The directionality of average treatment effects was maintained across both cohorts. While traditional group analysis may overlook important individual differences, the ITE identifies these variations to have more personalized treatment recommendations.

**Table 2:** Baseline characteristics of patients in UCSD and UCI Post-T0 initial HFNC/NIV cohorts by the predicted individualized treatment effect

Variable	UCSD (development site)				UCI (validation site)			
	NIV preferred	HFNC preferred	Indifferent	*P*^b^ value	NIV preferred	HFNC preferred	Indifferent	*P* value
**Characteristic**								
Encounters, N	1356	409	191	-	50	20	99	-
Age(years), mean (SD)	62(16.6)	62(16.4)	63(16.2)	0.028	61(14.5)	65(20.1)	65(17.7)	0.947
**Gender, N (%)**								
Male	824(60.8)	245(59.9)	106(55.5)	-	34(68.0)	11(55.0.0)	61(61.6)	-
**Organ dysfunction**								
**Charlson Comorbidity Index, Median (IQR)**	2.0(1.0-4.0)	3.0(1.0-5.0)	3.0(1.0–6.0)	0.049	2.5(1.0-5.5)	2.0(1.0-3.0)	2.0(0.5-3.0)	0.358
Congestive Heart Failure Component, N (%)	361(26.6)	125(30.6)	74(38.7)	0.692	30(30.3)	9(18.0)	5(25.0)	0.269
Chronic Obstructive Pulmonary Disease, N (%)	264(19.5)	85(20.8)	46(24.1)	0.585	7(7.1)	4(8.0)	1(5.0.)	0.907
SOFA score (at Vent.io T0), Median (IQR)	1.0(0.0-3.0)	1.0(0.0-3.0)	1.0(0.0-3.0)	0.627	1.0(0.0-3.0)	1.5(1.0-4.0)	1.0(0.0-3.0)	0.330
**Interventions after Vent.io T0**^a^ **N (%)**								
Steroids administration	426(31.4)	106(25.9)	50(26.2)	0.267	24(48.0)	10(50.0)	55(55.6)	0.945
Antibiotics administration	1100(81.9)	337(82.4)	151(79.1)	0.484	43(86.0)	19(95.0)	74(74.7)	0.031
Vasopressors administration	413(30.5)	130(31.8)	53(27.7)	0.219	15(30.0)	6(30.0)	29(29.3)	0.673
Diuretics administration	769(56.7)	240(58.7)	109(57.1)	0.049	24(48.0)	10(50.0)	55(55.6)	0.231
**Initial respiratory support after Vent.io T0 (N %)**								
HFNC	972(71.7)	274(67.0)	119(62.3)	-	38(76.0)	15(75.0)	78(78.8)	-
**Outcomes (N %)**								
IMV	337(24.9)	90(22.0)	29(15.2)	<0.001	22(37.9)	16(32.0)	3(15.0)	0.353
Mortality	421(31.0)	107(26.2)	50(26.2)	0.682	9(18.0)	4(20.0)	25(25.3)	0.582
Hospice	6(1.5)	11(0.8)	0(0.0)	0.590	14(28.0)	1(5.0)	21(21.2)	0.105
**Average treatment effect, mean (SD)**	-0.022 (0.023)	0.007 (0.007)	0.000 (0.0006)	0.022	-0.054 (0.098)	0.003 (0.001)	0.000 (0.0001)	<0.001

^a^ We focused on interventions administered from Vent.io T0 to 1 h before ICU discharge for the control group (those not intubated), and from Vent.io T0 to 1 h before the time of intubation for the positive group (those who require intubation).

^b^ Testing for the difference *P* value were χ2 for categorical variables and Kruskal-Wallis rank-sum test for continuous variables.

### SHAP-based feature interpretation of ITE predictions

**Fig. 1:**
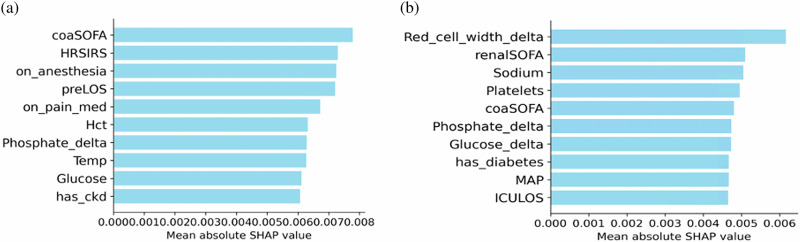
Top 10 features ranked by mean absolute SHAP value for ITE prediction using the RepFlow-CFR model. **a** UCSD site. **b** UCI site. *_delta is the short-term slope per hour between the last two non-missing values prior to T0 (Δt capped at 24 h). Complete feature definitions are provided in the Supplementary Table S6 (SHAP Feature Glossary for Fig. 1).

To interpret individualized treatment-effect (ITE) predictions from RepFlow-CFR, we computed SHAP values and ranked features by mean absolute contribution. Figure 1 shows the top 10 features for UCSD and UCI. At UCSD (Fig. 1a), severity and care-context variables, e.g., coaSOFA, HRSIRS, preLOS (pre-ICU hospital length of stay), and recent anesthesia/analgesic use, were most influential, suggesting that baseline acuity and peri-procedural context shaped recommendations. At UCI (Fig. 1b), laboratory measures and organ-dysfunction markers, particularly Red_cell_width_delta, renalSOFA, and Sodium, were more dominant, indicating site-specific patterns in model attribution. Several features (e.g., coaSOFA, Phosphate_delta) appeared in both cohorts, but their rankings differed, underscoring the influence of local data characteristics on treatment-effect predictions.

### Outcomes with treatment concordance

Our primary outcome was the need for invasive mechanical ventilation (IMV); the secondary outcome was a composite of in-hospital mortality or discharge to hospice. For each ITE method, we computed outcome proportions stratified by concordance: whether the first post-T0 respiratory support (HFNC or NIV) matched the modality preferred by the ITE model for that encounter (1 = matched; 0 = otherwise). Although hourly time-stamped data informed predictors, T0, and outcome timing, concordance itself was not evaluated hourly; later modality switches did not change the encounter-level concordance label.

**Fig. 2:**
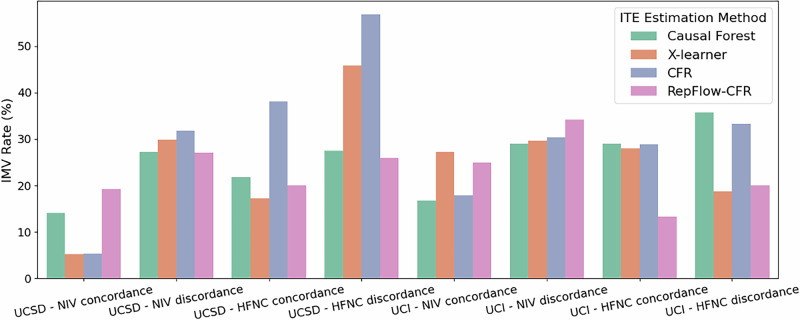
Comparison of IMV outcomes by treatment concordance and ITE estimation method. Bars represent IMV rates across four treatment groups (NIV concordance, NIV discordance, HFNC concordance, HFNC discordance) for each ITE method.

**Fig. 3:**
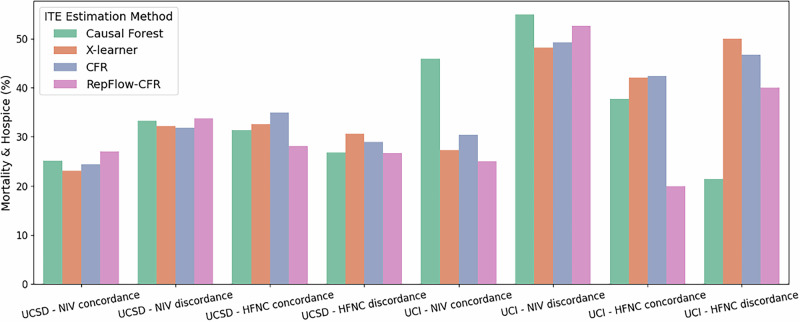
Comparison of Mortality & Hospice outcomes by treatment concordance and ITE estimation method. Bars represent Mortality & Hospice rates across four treatment groups (NIV concordance, NIV discordance, HFNC concordance, HFNC discordance) for each ITE method.

As illustrated in Figs. 2–3, outcomes were generally more favorable for patients who received concordant treatment across both sites. For IMV, RepFlow-CFR consistently showed lower rates in the concordant groups (e.g., UCSD/NIV: 19.27% vs 27.06%; UCI/HFNC: 13.33% vs 20.00%). Most methods exhibited similar trends, though the X-Learner at UCI showed a modestly higher IMV rate in the HFNC-concordant group. Mortality-and-Hospice results also reflected a concordance benefit with RepFlow-CFR (e.g., UCSD/NIV: 27.06% vs 33.74%; UCI/HFNC: 20.00% vs 40.00%). Overall, concordant treatment was associated with better outcomes, with RepFlow-CFR demonstrating the most consistent improvements across sites and outcomes.

To evaluate independence from confounding, we fit two multivariable logistic regression models: one for IMV and one for Mortality-and-Hospice, adjusting for age, sex (female = 1), SOFA at T0, Charlson Comorbidity Index (CCI), and Vent.io risk at T0. As shown in Table 3, concordance with the treatment recommended by RepFlow-CFR was significantly associated with reduced IMV risk at UCSD for both NIV and HFNC; in the composite model, RepFlow-CFR showed the strongest effect at UCI, with concordant NIV and concordant HFNC each significantly associated with lower Mortality-and-Hospice. These associations were not consistently observed with baseline recommenders (Causal Forest, X-Learner, CFR). Model discrimination (AUC) for all site-, outcome-, and engine-specific models is reported in Supplementary Table S11.

**Table 3:** Multivariable Logistic Regression Results (Odds Ratios and *p*-values) for Predicting the Need for IMV Across Methods and Sites

Method	Site	NIV concordance	HFNC concordance	Age	Gender	CCI score	SOFA score	Vent.io score
Causal Forest	UCSD	**0.443** ***p*** < **0.001**	**0.778** ***p*** = **0.033**	0.988 *p* < 0.001	1.046 *p* = 0.685	0.929 *p* < 0.001	1.044 *p* = 0.101	1.276 *p* = 0.083
	UCI	0.495 *p* = 0.245	0.896 *p* = 0.783	0.994 *p* = 0.559	1.046 *p* = 0.903	1.089 *p* = 0.165	1.051 *p* = 0.494	1.032 *p* = 0.949
X-learner	UCSD	**0.109** ***p*** < **0.001**	**0.356** ***p*** < **0.001**	0.984 *p* < 0.001	1.035 *p* = 0.769	0.932 *p* < 0.001	1.066 *p* = 0.021	1.267 *p* = 0.105
	UCI	0.495 *p* = 0.245	0.897 *p* = 0.783	0.992 *p* = 0.458	1.042 *p* = 0.912	1.069 *p* = 0.276	1.038 *p* = 0.606	1.015 *p* = 0.977
CFR	UCSD	**0.184** ***p*** < **0.001**	1.812 *p* < 0.001	0.992 *p* = 0.033	1.039 *p* = 0.745	0.949 *p* = 0.011	1.020 *p* = 0.475	1.294 *p* = 0.077
	UCI	0.495 *p* = 0.245	0.897 *p* = 0.783	0.992 *p* = 0.458	1.042 *p* = 0.912	1.069 *p* = 0.276	1.038 *p* = 0.606	1.015 *p* = 0.977
RepFlow-CFR	UCSD	**0.661** ***p*** = **0.0049**	**0.677** ***p*** = **0.019**	0.988 *p* < 0.001	1.003 *p* = 0.980	0.933 *p* < 0.001	1.047 *p* < 0.089	1.143 *p* < 0.350
	UCI	0.482 *p* = 0.373	0.243 *p* = 0.121	0.978 *p* = 0.252	0.554 *p* = 0.328	0.988 *p* = 0.901	1.092 *p* = 0.424	1.404 *p* = 0.747

The bold values correspond to statistically significant results, specifically when *p* < 0.05.

## Discussion

In this retrospective study of ICU patients at elevated risk for invasive mechanical ventilation (IMV), we developed and evaluated RepFlow-CFR, a deep counterfactual framework for estimating ITEs between HFNC and NIV as the initial post-trigger support. Across two academic centers, treatment concordance,alignment between the model’s preferred modality at the Vent.io T0 decision point and the first modality actually administered, was associated with more favorable outcomes for IMV and for a composite of in-hospital mortality or hospice. We interpret these findings as associations and explicitly do not claim causality.

Our design emphasizes temporality and clinical actionability. We used Vent.io to define T0, the moment a patient first crossed a predefined high-risk threshold for near-term IMV, then examined the first HFNC/NIV administered thereafter. Concordance was defined prospectively at T0 from the ITE-preferred modality and analyzed at the encounter level. Multivariable logistic models adjusted for a prespecified baseline set (age, sex, SOFA at T0, Charlson Comorbidity Index, and Vent.io risk at T0) to account for measured differences in acuity and comorbidity. Analyses were conducted separately for UCSD and UCI to respect institutional differences in practice.

Several considerations temper interpretation. First, the “high-risk” cohort is prediction-defined: we applied a single Vent.io alert threshold selected during development to target ~ 60% encounter-level sensitivity for 24-h IMV. This choice enriches the cohort for patients in whom escalation decisions are consequential, but it does not correspond to clinical severity bands and introduces heterogeneity by design. Given the underlying event rate at this operating point, positive predictive values near ~0.17 are expected and should not be taken to imply bias toward low-severity patients. Thresholding may also miss some eventual IMV cases that deteriorate rapidly after T0, and results may not generalize outside the model-flagged population.

Second, concordance is susceptible to residual confounding. Although we adjusted for key baseline factors, unmeasured variable such as patient tolerance (e.g., mask intolerance, secretion burden) and institutional preferences could influence both the initial device and outcomes, especially given site-level differences. For this reason, we avoided post-hoc subgrouping or additional robustness analyses in this revision and frame concordance findings as hypothesis-generating.

Third, external generalizability merits caution. Discrimination declined when the UCSD-tuned models were applied “as is” at UCI, then improved with site-specific fine-tuning on a random 25% subset. The UCI sample size is modest, and method-to-method differences varied by site (e.g., CFR’s competitive performance at UCI), so conclusions are best described as consistent trends rather than universal superiority.

Fourth, we did not examine duration-based endpoints (e.g., ventilator-free days). Because concordance can affect the probability of intubation, restricting analysis to intubated patients risks selection (collider) bias, and post-intubation duration is influenced by downstream practices (sedation, weaning, tracheostomy, goals-of-care). We now list this as a limitation and pre-specify ventilator-free days to day 28 and ICU-free days for prospective evaluation using selection-aware time-to-event methods.

Methodologically, RepFlow-CFR integrates representation learning with conditional normalizing flows to model outcome distributions and to adjust for hidden confounding signals that can arise from clinician decision-making and unobserved severity. We adopted a conservative indifference band for ITE classification (|ITE | ≤ 0.001, i.e., ≤ 0.1 percentage-point absolute risk difference) to buffer near-zero estimation noise and discourage action on clinically trivial differences. We also addressed EHR missingness by incorporating time-since-last-measurement (TSLM) with learnable gating; nonetheless, Missing Not at Random (MNAR) mechanisms may persist and are acknowledged as a limitation.

These results suggest that individualized recommendations at a clearly defined deterioration trigger may complement guideline-informed practice where evidence is conditional or mixed. In settings with competing indications and contraindications for HFNC versus NIV, counterfactual estimates could help identify patients more likely to benefit from one modality over the other and highlight patients for whom the modalities appear interchangeable (indifferent band).

Establishing causal impact will require prospective, clinician-in-the-loop evaluation (e.g., pragmatic or stepped-wedge designs) that (i) prespecify operating thresholds and alert burden, (ii) incorporate duration-based endpoints (VFD-28, ICU-free days), and (iii) assess calibration, decision-curve net benefit, and workflow effects. Broader validation across community and international sites, as well as comparisons across alternative operating points and model families (e.g., CFR vs RepFlow-CFR), will further clarify external performance and implementation considerations.

In summary, concordance between model recommendations and the initial post-T0 modality was associated with improved outcomes across two centers, with site-specific variation. Given the observational design, prediction-defined cohort, and residual confounding, these findings should be viewed as hypothesis-generating. Prospective testing will be essential to determine whether model-guided selection of HFNC versus NIV can causally improve patient outcomes.

## Methods

### Study design and setting

We conducted a retrospective study using de-identified Electronic Health Record (EHR) data of all adult patients (≥18 years) who were admitted to the ICU at University of California San Diego Health (UCSD) between January 1, 2016, and December 31, 2023, and the University of California Irvine Health (UCI) between January 1, 2021, and August 15, 2022, as well as January 1, 2023, and August 31, 2024. This study was approved by the Institutional Review Boards of the University of California San Diego (IRB# 800123). Given the retrospective design and use of de-identified data, the requirement for informed consent was waived by both IRBs.

Patients were included in the respiratory failure prediction analysis if they had an ICU stay of at least 5 h, were not mechanically ventilated before ICU admission, and had recorded measurements of heart rate, blood pressure, and laboratory values prior to the prediction start time. If there were multiple admissions for a single patient, each ICU stay was treated as a separate encounter. Patients with a “Do Not Resuscitate” (DNR) order were excluded, and timestamps within 24 h before and after surgery were omitted to avoid capturing surgery-related ventilation events. Monitoring for respiratory failure continued throughout each ICU stay until either mechanical ventilation was initiated or the patient was transferred out of the ICU. To ensure sufficient data collection, predictions began 4 h after ICU admission and were updated hourly using the latest clinical data.

We used the pretrained Vent.io respiratory failure risk prediction model to identify high-risk ICU patients by thresholding the predicted state value. The model was trained using a custom 5-point labeling scale for mechanical ventilation (Table S1) to account for the various physiological states of respiratory failure, where a score ≥ 3 was defined as positive class and <3 was control class. The high-risk threshold was selected at 60% sensitivity at the encounter level of the training data, based on clinical feedback, to minimize false positives. This threshold was then applied during model inference: if the predicted score met or exceeded the threshold, the patient was classified as high-risk for requiring IMV within the next 24 h. A score below the threshold indicated that IMV was not predicted within the next 24 h.

**Fig. 4:**
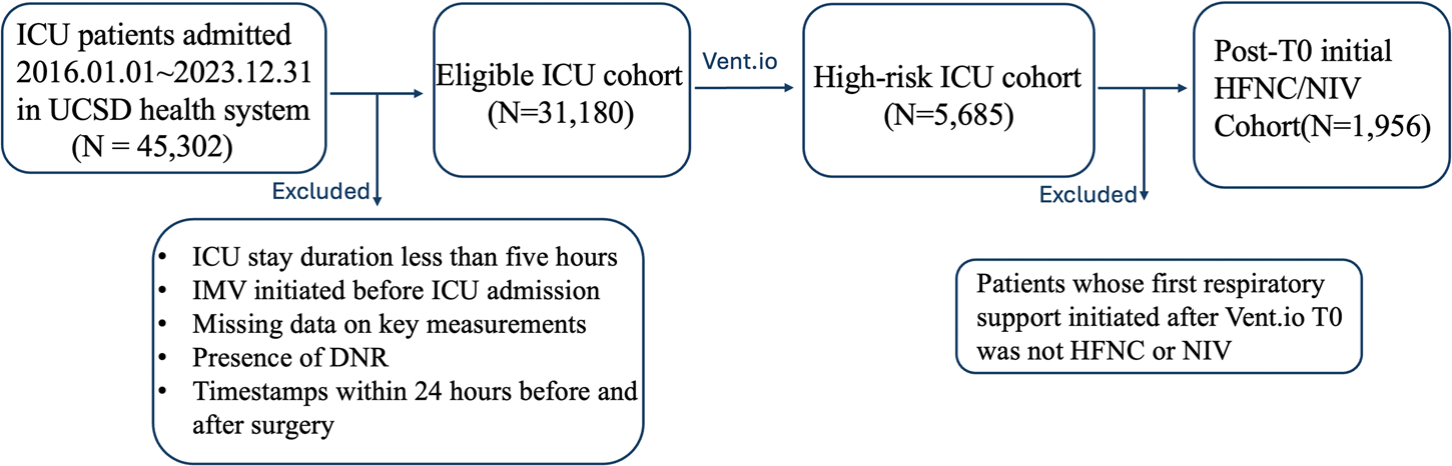
Cohort selection flowchart for Post-T0 initial HFNC/NIV analysis using UCSD cohort as an example. The flowchart shows patient selection from 45,302 ICU admissions to the final cohort of 1,956 patients, with exclusion criteria listed in the boxes.

To evaluate the effect of initial treatment of HFNC and NIV on high-risk ICU patients, we defined Vent.io T0 as the time at which the patient’s risk score first crossed the high-risk threshold (score ≥ 3). We focused on the clinical data at Vent.io T0 and the initial respiratory support treatment administered after this timepoint, specifically including patients who first received either HFNC or NIV (referred to as Post-T0 initial HFNC/NIV cohort). Treatment categories were further refined to distinguish specific HFNC and NIV interventions (Table S2). Figure 4 illustrates the cohort derivation process for the analysis of early HFNC and NIV as initial respiratory support following Vent.io-predicted high-risk timepoint (Vent.io T0). Using counterfactual modeling, we aimed to compare the effects of HFNC and NIV on the subsequent need for IMV and mortality.

### Data abstraction, missingness, and processing

We extracted data from EHR including 50 vital signs and laboratory measurements, 6 demographic features, 12 Systemic Inflammatory Response Syndrome (SIRS) and Sequential Organ Failure Assessment (SOFA) criteria, 12 medication categories, and 62 comorbidities (Table S3). The vital signs and laboratory measurements were grouped into one-h time series bins to account for varying data sampling frequencies. Variables sampled more than once per hour were resampled into hourly bins using their median. Updates were made hourly with new data and if no new data were present, existing values were carried forward for up to 24 h. All remaining missing values were replaced using mean imputation. We reported missing data on an hourly interval following the resampling process for the 50 vital signs and laboratory measurements (Table S4). In addition to the above clinical variables, we calculated 150 features derived from the 50 vital signs and laboratory measurements. For each vital sign and laboratory measurement, we derived baseline values (mean value measured over the previous 72 h), local trends (change since last measurement), and the time since the variable was last measured (TSLM). Table 4

**Table 4:** Multivariable Logistic Regression Results (Odds Ratios and *p*-values) for Predicting Mortality & Hospice Across Methods and Sites

Method	Site	NIV concordance	HFNC concordance	Age	Gender	CCI score	SOFA score	Vent.io score
Causal Forest	UCSD	**0.663** ***p*** = **0.009**	1.040 *p* = 0.723	1.020 *p* < 0.001	0.844 *p* = 0.104	0.955 *p* = 0.010	1.260 *p* < 0.001	1.245 *p* < 0.100
	UCI	0.409 *p* = 0.089	0.731 *p* = 0.397	1.014 *p* = 0.155	0.706 *p* = 0.296	0.984 *p* = 0.775	1.092 *p* = 0.202	2.086 *p* = 0.120
X-learner	UCSD	**0.592** ***p*** < **0.001**	1.034 *p* = 0.775	1.021 *p* < 0.001	0.855 *p* = 0.136	0.959 *p* = 0.019	1.251 *p* < 0.001	1.249 *p* < 0.097
	UCI	**0.409** ***p*** = **0.089**	0.731 *p* = 0.397	1.011 *p* = 0.239	0.723 *p* = 0.337	0.971 *p* = 0.622	1.094 *p* = 0.199	2.329 *p* = 0.079
CFR	UCSD	**0.669** ***p*** = **0.003**	1.190 *p* = 0.184	1.022 *p* < 0.001	0.831 *p* = 0.080	0.960 *p* = 0.026	1.237 *p* < 0.001	1.274 *p* = 0.071
	UCI	0.409 *p* = 0.089	0.731 *p* = 0.397	1.011 *p* = 0.239	0.723 *p* = 00.337	0.971 *p* = 0.622	1.094 *p* = 0.199	2.329 *p* = 0.079
RepFlow-CFR	UCSD	**0.679** ***P*** = **0.0049**	0.749 *P* = 0.063	1.020 *P* < 0.001	0.884 *P* = 0.265	0.953 *P* = 0.011	1.278 *P* < 0.001	1.213 *P* = 0.160
	UCI	**0.092** ***P*** = **0.020**	**0.088** ***P*** = **0.012**	1.021 *P* = 0.292	0.726 *P* = 0.608	1.103 *P* = 0.295	1.368 *P* = 0.010	3.027 *P* = 0.276

The bold values correspond to statistically significant results, specifically when *p* < 0.05.

### Model development, training and evaluation

**Fig. 5:**
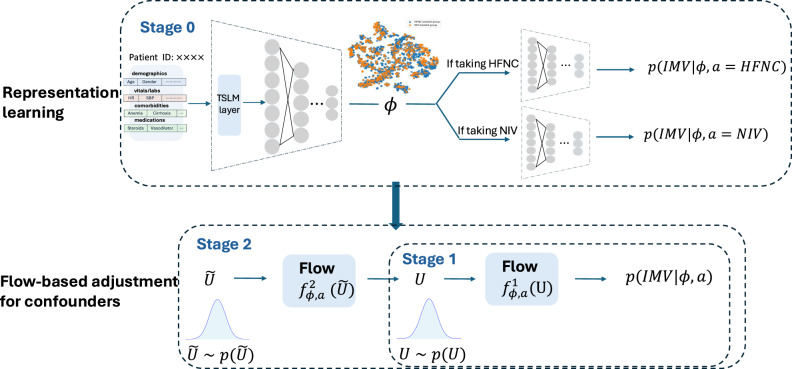
Overview of the RepFlow-CFR model architecture, showing three stages. (Stage 0) representation learning using patient features to generate the representation; (Stage 1) output distribution modeling for IMV prediction; and (Stage 2) flow-based confounder adjustment using normalizing flows.

We proposed the RepFlow-CFR model, a flow-based confounder adjustment model that integrates representation learning, normalizing flows and counterfactual inference. Figure 5 presents the architecture overview of the RepFlow-CFR model, which contains three modules as follows.

Stage 0 (CFR-based representation learning): We utilized the counterfactual regression architecture (CFR)^17^ that includes shared representation layers and two distinct heads for predicting outcomes under different treatments. The shared representation layers, based on the Vent.io architecture, include a TSLM layer for adjusting the importance of labs and vitals, followed by a feedforward neural network. By training the CFR model, the shared representation is encouraged to balance the distribution of measured confounders across treatment groups. The loss function is formulated as

1 $${L}_{0}=E\left[{L}_{{CFR}}\left(x,a,y\right)+\lambda \cdot {IP}{M}_{G}\left({\left\{{\phi }_{i}\right\}}_{i,a={HFNC}},{\left\{{\phi }_{i}\right\}}_{i,a={NIV}}\right)\right],$$

where ${IP}{M}_{G}$ is the empirical probability metric (e.g. Wasserstein distance). ${L}_{{CFR}}$ is the prediction loss. $\lambda$ denotes the trade-off parameter that balances prediction accuracy and representation distribution matching.

After training Stage 0, we assume that the learned representation $\phi$ captures sufficient information from measured covariates such that potential outcomes are conditionally independent of treatment assignment, given $\phi$ and a latent variable $u$ representing unmeasured confounding as

2 $$Y\left(A\right)\coprod A\,|\phi ,u,$$

where $Y$ and $A$ denote the outcome and treatment, respectively.

The observed distribution is defined as

3 $$p\left(y|\phi ,\,a\right)=\int p({y|}\phi ,u,a)p({u|}\phi ,a){du}.$$

The interventional distribution, which removes the influence of $a$ on $u$, is

4 $$p\left(Y\left(a\right)=y|\phi \right)=\int p({y|}\phi ,u,a)p({u|}\phi ){du}.$$

If $p\left(u|\phi ,\,a\right)=p{(}{u}{|}{\phi }{)},$ we will have $p\left(Y\left(a\right)=y|\phi \right)=p\left(y|\phi ,\,a\right)$. However, in observational studies, this assumption rarely holds due to treatment assignment bias or unmeasured confounding. Directly using $p\left(y|\phi ,\,a\right)$ to estimate counterfactual outcomes would lead to biased inference. To address this, RepFlow-CFR includes two additional stages to explicitly model and account for this hidden bias.

Stage 1 (Modeling outcome distribution): We used a conditional normalizing flow (CNF)^21^ to model the observed outcome distribution $p\left(y|\phi ,\,a\right)$, where $a\in \left\{{NIV},{HFNC}\right\}$. The CNF learns an invertible transformation ${f}_{{\rm{\phi }},{\rm{a}}}^{1}$ that maps a standard normal latent variable $U \sim N(0,I)$ to the outcome space. The loss function is formulated as

5 $${L}_{1}=\mathop{\sum }\limits_{i=1}^{n}p\left({f}_{{\phi }_{i},{a}_{i}}^{1}\left(U\right)={y}_{i}\right).$$

Stage 2 (Adjusting for hidden confounding): Since the learned representation $\phi$ may not satisfy unconfoundedness due to unmeasured confounding, we introduced a second CNF ${f}_{{\rm{\phi }},{\rm{a}}}^{2}$. It transforms a new latent variable $\widetilde{U} \sim N(0,I)$ to an interventional latent variable that ~$p({u|}\phi )$. The resulting latent sample is then passed through the Stage 1 transformation

${f}_{{\rm{\phi }},{\rm{a}}}^{1}$, which shifts the latent distribution $p\left(u,|,\phi ,a\right)$ toward the interventional distribution $p({u|}\phi )$. This adjustment allows us to account for hidden biases arising from factors like clinician decision-making, treatment selection bias, and unobserved patient severity. The loss function is formulated as

6 $${L}_{2}=\mathop{\sum }\limits_{i=1}^{n}p\left({f}_{{\phi }_{i},{a}_{i}}^{1}\left({f}_{{\phi }_{i},{a}_{i}}^{2}(\widetilde{U})\right)={y}_{i}\right).$$

Inference stage: During inference, we sampled $\widetilde{U}$ using the CNF from Stage 2 and mapped it to ${y}$ using the CNF from stage 1, then averaged multiple predicted outcomes. ITE was obtained by the difference between the averaged outcomes under NIV and under HFNC.

We split the UCSD Post-T0 HFNC/NIV cohort into 80% training and 20% internal validation, using the 20% set exclusively for hyperparameter tuning and early stopping. Hyperparameters (learning rate, weight decay, hidden layers/width, flow depth, batch size, IPM weight) were selected via a fixed grid (Supplementary Table S12) with Bayesian optimization^22^. All modules were trained with Adam optimizer^23,24^ and early stopping. Stages were trained separately (CFR backbone → outcome CNF → latent-adjustment CNF). For external adaptation, we warm-started from the UCSD-tuned checkpoint and fine-tuned on a random stratified 25% subset of the UCI Post-T0 cohort, reusing the UCSD-selected hyperparameters with no additional search.

Model performance, predictive accuracy and ITE estimation quality, was evaluated on the held-out UCSD validation set and externally on the independent UCI Post-T0 HFNC/NIV cohort. After hyperparameter selection, the model was retrained on the UCSD 80% training subset and then applied to the full UCSD cohort to compute ITEs for descriptive cohort-level summaries. For UCI, we first evaluated the frozen UCSD-tuned model and then, in a prespecified secondary analysis, fine-tuned CFR and RepFlow-CFR on a random 25% of UCI. Finally, we applied the fine-tune model to the whole UCI to compute ITE.

The predicted ITE was defined as the difference between the predicted probability of IMV under NIV and under HFNC, when each was given as the first intervention following the Vent.io T0 timepoint. To assess predictive performance, we reported two standard metrics for IMV prediction: the Area Under the Receiver Operating Characteristic Curve (AUC) and the Area Under the Precision-Recall Curve (PR-AUC). ITE estimation quality was further evaluated by examining patient outcomes under treatment concordance. As baseline comparisons, we included commonly used data-driven ITE estimation methods in clinical settings, including Causal Forest^14^, X-Learner^15^, and the CFR model.

## Supplementary information

Supplementary information

## Data Availability

Data supporting the findings of this study can be provided upon reasonable request to the corresponding author.

## Code availability

Code used in this study can be made available upon reasonable request to the corresponding author.
