# Supplementary material for: Comparing high-flow nasal cannula and non-invasive ventilation in critical care: insights from deep counterfactual inference
Source: Npj Health Syst. 2025 Dec 4;2:45. doi: 10.1038/s44401-025-00049-w (PMC12678170; doi:10.1038/s44401-025-00049-w)
Supplement: Supplementary file 1 — Supplementary information [file 44401_2025_49_MOESM1_ESM.docx]

**Supplementary Materials for “Comparing High-Flow Nasal Cannula and Non-Invasive Ventilation in Critical Care: Insights from Deep Counterfactual Inference”**

Supplementary Table 1. Criteria of clinical labeling scheme of Vent.io model.

| **Condition** | **Criteria** | **Points** |
| --- | --- | --- |
| PaO2/FiO2 (not NaN) | 200$\leq$PaO2/FiO2$\leq$300 mmHg | 1 |
|  | PaO2/FiO2 $\leq$ 200 mmHg (severe hypoxemia) | 2 |
|  | IMV $\leq$ 24 hours | 3 |
|  | PaO2/FiO2 $\leq$200 mmHg and IMV <=24 hours | 4 |
|  | IMV > 24 hours or (IMV $\leq$ 24 hours + mortality) | 5 |
| SpO2/FiO2 (not NaN) | 141$\leq$PaO2/FiO2$\leq$221 mmHg | 1 |
|  | SpO2/FiO2 $\leq$ 141 mmHg (severe hypoxemia) | 2 |
|  | IMV $\leq$ 24 hours | 3 |
|  | SpO2/FiO2 $\leq$141 mmHg and IMV $\leq$24 hours | 4 |
|  | IMV > 24 hours or (IMV $\leq$24 hours + mortality) | 5 |

Supplementary Table 2. Categories of HFNC and NIV treatments used in the study cohorts.

| Category | Measurement name |
| --- | --- |
| HFNC | High Flow Nasal Cannula  High Flow Mask  Heated High Flow Blender  Neb High Flow  Head Hood;High Flow Nasal Cannula  Aerosol mask;High Flow Mask  Oxyhood;Heated High Flow blender  High Flow Mask;Heated High Flow Blender  High Flow Nasal Cannula;Heated High Flow Blender  Aerosol Mask;Neb High Flow  Simple mask;Heated High Flow Blender  Heated High Flow Blender;Other (Comment)  Heated High Flow Blender;High Flow Nasal Cannula  Non-rebreather mask;Heated High Flow Blender  Heated High Flow Blender;Neb High Flow  Heated High Flow Blender;NC  Heated High Flow Blender;Head Hood  Heated High Flow Blender;Non-rebreather mask  Neb High Flow;Aerosol Mask  Venturi Mask;Heated high Flow Blender  Heated High Flow Blender;High Flow Mask  Heated High Flow Blender; heated High Flow Blender |
| NIV | BIPAP  CPAP  Bag Valve Mask |

Supplementary Table 3. Model features summary.

| **Demographic features** | Age, Gender, care unit identifier - ED, care unit identifier- Ward, hours between hospital admit and care unit admit (ED/Ward), duration from care unit admission until current time |
| --- | --- |
|  |  |
| **Vital signs and laboratory measurements** | HR, O2Sat, Temp, SBP, MAP, DBP, Resp, EtCO2, BaseExcess, HCO3, FiO2, pH, PaCO2, SaO2, AST, BUN, Alkalinephos, Calcium, Chloride, Creatinine, BilirubinDirect, Glucose, Lactate, Magnesium, Phosphate, Potassium, BilirubinTotal, TroponinI, Hct, Hgb, PTT, WBC, Fibrinogen, Platelets, PaO2, BNP, Sodium, Procalcitonin, CRP, Ammonia, Hgb_A1C, lymphocytes, lymphocytes_differential, albumin, ALT, D_Dimer, Ferritin, Red_cell_width, Sedimentation_Rate, LDH |
| **Comorbidities** | Disseminated intravascular coagulation, Necrotizing Fasciitis, Tumor Lysis Syndrome, Shock, Metastatic Cancer, STEMI, Myeloblastic Leukemia, Liver Failure, Malignant Pleural Effusion, Pneumothorax, Cachexia, Coma, Peritonitis, Lymphoma, Endocarditis, Myocarditis, Pericarditis, Septic Embolism, Coagulation Defect, Primary Lung Cancer, Lymphoid Leukemia, Pericardial Effusion, Cerebral Infarction, Meningitis/Encephalitis, Malignant Liver Cancer, GI Hemorrhage, Acute Renal Failure, Chronic Liver Disease, Neutropenia, Cardiac Arrhythmia, Malnutrition, Pulmonary Heart Disease, Chronic Kidney Disease, Plasma Protein Disorder, Pancytopenia/Aplasia, Aspiration Pneumonitis, AMI/Non-STEMI, CHF, Encephalopathy, Thrombocytopenia Including Purpuric, HIT, & Other Platelet defects, Coronary artery disease, COPD, Diabetes, HIV, Hypertension, Obesity, Severe Brain Conditions, Other Pulmonary Conditions, Non-Rheumatic Valve Disease, UTI, Anemia, Pneumonia, Hyperlipidemia, Major depressive disorder, Bacterimia, Gastro-esophageal reflux disease, Hypothyroidism, Long term use of insulin, Liver cirrhosis, Immune conditions, Solid malignancy, Organ transplant, Rheumatologic/Inflammatory |
| **Medications** | Anesthesia, Anticoagulants, Anticonvulsants, Antipsychotics, Bleeding reversal agents, Continuous infusion vasodilator, Continuous infusion anti-hypertensive, Continuous infusion antiarrhythmics, Inhaled prostacyclin, Continuous infusion for neuromuscular blockade, Continuous infusion for pain, Steroids |

Supplementary Table 4. Comparison of missing data in two ICU cohorts.

| **Feature** | **UCSD (%)** | **UCI (%)** |
| --- | --- | --- |
| HR | 0.004 | 0.002 |
| O2Sat | 0.02 | 0.003 |
| Temp | 0.25 | 0.05 |
| SBP | 5.05 | 0.08 |
| MAP | 5.28 | 0.11 |
| DBP | 5.05 | 0.08 |
| Resp | 0.11 | 0.06 |
| EtCO2 | 89.91 | 97.36 |
| BaseExcess | 83.08 | 78.46 |
| HCO3 | 6.12 | 100.00 |
| FiO2 | 88.90 | 82.60 |
| pH | 82.88 | 90.94 |
| PaCO2 | 83.19 | 91.08 |
| SaO2 | 83.46 | 81.23 |
| AST | 53.55 | 47.36 |
| BUN | 5.97 | 12.30 |
| Alkalinephos | 53.50 | 47.10 |
| Calcium | 5.97 | 12.30 |
| Chloride | 5.99 | 12.37 |
| Creatinine | 6.02 | 12.49 |
| BilirubinDirect | 85.50 | 70.21 |
| Glucose | 5.96 | 12.37 |
| Lactate | 75.37 | 66.80 |
| Magnesium | 19.10 | 17.75 |
| Phosphate | 24.69 | 21.10 |
| Potassium | 5.78 | 12.43 |
| BilirubinTotal | 54.33 | 47.23 |
| TroponinI | 84.94 | 100.00 |
| Hct | 7.91 | 13.44 |
| Hgb | 7.21 | 13.44 |
| PTT | 57.02 | 66.22 |
| WBC | 8.10 | 13.62 |
| Fibrinogen | 94.40 | 94.04 |
| Platelets | 8.08 | 13.77 |
| PaO2 | 83.08 | 91.02 |
| BNP | 94.51 | 100.00 |
| Sodium | 6.45 | 18.85 |
| Procalcitonin | 92.67 | 91.28 |
| CRP | 99.72 | 93.35 |
| Ammonia | 97.67 | 96.25 |
| Hgb_A1C | 89.93 | 89.94 |
| lymphocytes | 20.19 | 100.00 |
| lymphocytes_differential | 19.19 | 43.28 |
| albumin | 52.13 | 47.99 |
| ALT | 54.87 | 48.98 |
| D_Dimer | 98.31 | 96.39 |
| Ferritin | 98.01 | 94.33 |
| Red_cell_width | 8.10 | 13.49 |
| Sedimentation_Rate | 97.57 | 95.16 |
| LDH | 93.56 | 93.69 |

Supplementary 5: The Vent.io pretraining process.

The architecture of Vent.io was described in Supplementary Figure 1. For pretraining Vent.io UCSD ICU cohort was randomly split into 80% for training and 20% for validation. We employed Bayesian optimization on the training set to fine-tune hyperparameters, including learning rate, hidden layers, dropout rate, and weight decay. The model was trained using the fine-tuned hyperparameters, with early stopping applied to prevent overfitting by selecting the best checkpoint based on validation performance. The threshold was selected to achieve a sensitivity of 0.6 in the training set. On the validation set, using this threshold, the pretrained Vent.io achieved an AUC of 0.882, sensitivity of 0.588, specificity of 0.841, and positive predictive value of 0.170, demonstrating robust performance in predicting IMV need within 24 hours.


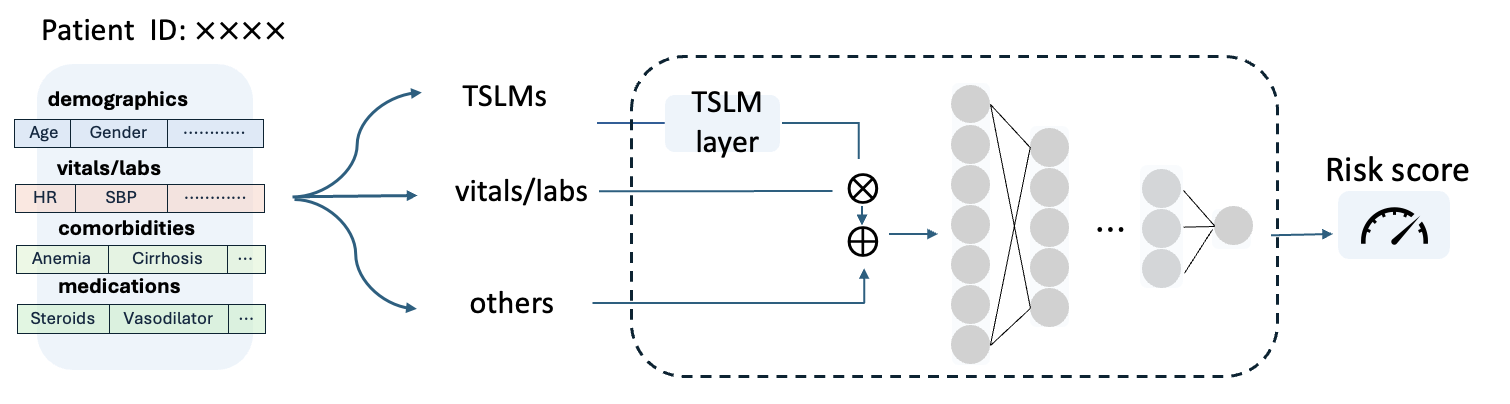


Supplementary Figure 1. Vent.io architecture, including a weighted layer (TSLM layer) for adjusting the importance of labs and vitals, followed by the feedforward neural network.

Supplementary Table 5. Patient characteristics for UCSD and UCI ICU cohorts.

| Variable | UCSD | | UCI | |
| --- | --- | --- | --- | --- |
|  | Control group | Positive group | Control group | Positive group |
| Encounters, N (%) | 29544(94.8) | 1636(5.2) | 3110(94.5) | 180(5.5) |
| Age (years), Median (IQR) | 62.2(49.1-73.2) | 61.9(50.3-71.9) | 61.4(44.9-72.7) | 63.0(51.8-73.7) |
| Gender, % |  |  |  |  |
| Male | 41.7 | 37.3 | 43.2 | 42.8 |
| Per Visit ICU Length of Stay (hours), Median (IQR) | 46.6(29.4-75.6) | 141.4(66.3-250.7) | 50.8(31.9-86.7) | 126.6(56.8-256.5) |
| CCI, Median (IQR) | 2.0(1.0-4.0) | 2.0(1.0-4.0) | 1.0(0.0-3.0) | 2.0(1.0-5.0) |
| SOFA, Median (IQR) | 2.0(1.0-4.0) | 9.0(7.0-12.0) | 2.0(1.0-3.0) | 10.0(6.0-12.0) |
| Mortality, % | 5.7 | 38.8 | 3.3 | 22.2 |
| Time from ICU admission to initiation of MV (hours), Median (IQR) | - | 26.9(11.0-58.0) | - | 28.0(11.0-57.0) |

Supplementary Table 6. Definitions of features in the SHAP plots.

| **Feature** | **Definition** |
| --- | --- |
| **coaSOFA** | SOFA coagulation subscore |
| **renalSOFA** | SOFA renal subscore |
| **HRSIRS** | Number of SIRS criteria (HR, RR, Temp, WBC) met |
| **preLOS** | Hours from hospital admission → ICU admission |
| **ICULOS** | Hours from ICU admission → T0. |
| **on_anesthesia** | Any anesthetic/sedative |
| **on_pain_med** | Any analgesic/opioid |
| **has_ckd** | Chronic kidney disease documented before ICU |
| **has_diabetes** | Diabetes documented before ICU |
| **Hct** | Hematocrit |
| **Temp** | Body temperature |
| **Glucose** | Blood glucose |
| **Sodium** | Serum sodium |
| **Platelets** | Platelet count |
| **MAP** | Mean arterial pressure |
| **Phosphate_delta** | Per-hour slope of serum phosphate between the last two non-missing values prior to T0 (Δt ≤ 24 h). |
| **Glucose_delta** | Per-hour slope of glucose between the last two non-missing values prior to T0 (Δt ≤ 24 h). |
| **Red_cell_width_delta** | Per-hour slope of RDW between the last two non-missing values prior to T0 (Δt ≤ 24 h). |

Supplementary Table 7. IMV Rates Stratified by Model Recommendation and Concordance (%).  (UCSD site)

| **Model** | **Recommendation** | **Total**  **IMV** | **Concordant**  **IMV** | **Discordant**  **IMV** |
| --- | --- | --- | --- | --- |
| **Causal Forest** | **NIV** | 22.98  (213 /927) | 14.04  (42/299) | 27.22  (171/628) |
|  | **HFNC** | 23.44  (240/1024) | 21.83  (160/733) | 27.49  (80/291) |
| **X-Learner** | **NIV** | 21.98  (251/1142) | 5.21  (19/365) | 29.86  (232/777) |
|  | **HFNC** | 25.25  (202/800) | 17.22  (99/575) | 45.78  (103/225) |
| **CFR** | **NIV** | 13.97  (184/1317) | 5.35  (22/141) | 17.88  (162/906) |
|  | **HFNC** | 43.60   (252/578) | 38.14  (156/409) | 56.80  (96/169) |
| **RepFlow-CFR** | **NIV** | 24.85  (337/1356) | 19.27  (74/384) | 27.06  (263/972) |
|  | **HFNC** | 22.00  (90/409) | 20.07  (55/274) | 25.93  (35/135) |

Supplementary Table 8. Mortality & Hospice Rates Stratified by Recommendation and Concordance (%). (UCSD site)

| **Model** | **Recommendation** | **Total**  **Mortality & Hospice** | **Concordant**  **Mortality & Hospice** | **Discordant**  **Mortality &**  **Hospice** |
| --- | --- | --- | --- | --- |
| **Causal Forest** | **NIV** | 30.64  (284 /927) | 25.08  (75/299) | 33.28  (209/628) |
|  | **HFNC** | 30.08  (308/1024) | 31.38  (230/733) | 26.80  (78/291) |
| **X-Learner** | **NIV** | 29.25  (334/1142) | 23.01  (84/365) | 32.18  (250/777) |
|  | **HFNC** | 32.00  (256/800) | 32.52  (187/575) | 30.67  (69/225) |
| **CFR** | **NIV** | 29.46  (388/1317) | 24.33  (100/411) | 31.79  (288/906) |
|  | **HFNC** | 33.22  (192/578) | 34.96  (143/409) | 28.99  (49/169) |
| **RepFlow-CFR** | **NIV** | 31.86  (432/1356) | 27.06  (104/304) | 33.74  (328/972) |
|  | **HFNC** | 27.63  (113/409) | 28.10  (77/274) | 26.67  (36/135) |

 Supplementary Table 9. IMV Rates Stratified by Model Recommendation and Concordance (%).  (UCI site)

| **Model** | **Recommendation** | **Total**  **IMV** | **Concordant**  **IMV** | **Discordant**  **IMV** |
| --- | --- | --- | --- | --- |
| **Causal Forest** | **NIV** | 25.58  (22/86) | 16.67  (4/24) | 29.03  (18/62) |
|  | **HFNC** | 30.12  (25 /83) | 28.99  (20/69) | 35.71  (5/14) |
| **X-Learner** | **NIV** | 43.69  (45/103) | 27.27  (6/22) | 29.63  (24/81) |
|  | **HFNC** | 43.94  (29/66) | 28.0  (14/50) | 18.75  (3/16) |
| **CFR** | **NIV** | 27.17  (25/92) | 17.39  (4/23) | 30.43  (21/69) |
|  | **HFNC** | 29.73  (22/74) | 28.81  (17/59) | 33.33  (5/15) |
| **RepFlow-CFR** | **NIV** | 32.0  (16/50) | 25.00  (3/12) | 34.21  (13/38) |
|  | **HFNC** | 15.0  (3/20) | 13.33  (2/15) | 20.00  (1/5) |

Supplementary Table 10. Mortality & Hospice Rates Stratified by Recommendation and Concordance (%). (UCI site)

| **Model** | **Recommendation** | **Total**  **Mortality & Hospice** | **Concordant**  **Mortality & Hospice** | **Discordant**  **Mortality &**  **Hospice** |
| --- | --- | --- | --- | --- |
| **Causal Forest** | **NIV** | 52.33  (45/86) | 45.83  (11/24) | 54.84  (34/62) |
|  | **HFNC** | 34.94  (29/83) | 37.68  (26/69) | 21.43  (3/14) |
| **X-Learner** | **NIV** | 29.13  (30/103) | 27.27  (6/22) | 23.46  (39/81) |
|  | **HFNC** | 25.76  (17/66) | 42.0  (21/50) | 50.0  (8/16) |
| **CFR** | **NIV** | 44.57  (41/92) | 24.63  (7/23) | 49.28  (34/69) |
|  | **HFNC** | 43.24  (32/74) | 42.37  (25/59) | 46.67  (7/15) |
| **RepFlow-CFR** | **NIV** | 46.0  (23/50) | 25.00  (3/12) | 52.63  (20/38) |
|  | **HFNC** | 25.0  (5/20) | 20.00  (3/15) | 40.00  (2/5) |

Supplementary Table 11. Discrimination (AUC %) of Multivariable Logistic Regression Models for IMV and Mortality/Hospice.

| **Methods** | **UCSD** | | **UCI** | |
| --- | --- | --- | --- | --- |
|  | **IMV** | **Mortality & Hospice** | **IMV** | **Mortality & Hospice** |
| **Causal Forest** | 61.33 | 65.86 | 61.84 | 61.13 |
| **X-Learner** | 70.82 | 65.99 | 59.85 | 65.28 |
| **CFR** | 68.68 | 65.69 | 61.40 | 64.63 |
| **RepFlow-CFR** | 60.08 | 66.48 | 63.78 | 80.53 |

Supplementary Table 12. Hyperparameter search space (Bayesian optimization).

| **Hyperparameter** | **Search space (from code)** | **Notes** |
| --- | --- | --- |
| **CFR: number of hidden layers** | {2, 3, 4} | sampled uniformly at each Bayesian optimization call |
| **CFR: hidden layer width** | {50, 55, 60, …, 150} | step = 5 (from 50–150) |
| **CFR: width decay factor** | {0.5, 0.7, 0.9} | derived from 0.5–0.9 with step 0.2 |
| **CNF (Stage-2): number of hidden layers** | {2, 3, 4} | sampled uniformly |
| **CNF (Stage-2): hidden layer width** | {50, 55, 60, …, 150} | step = 5 |
| **CNF (Stage-2): width decay factor** | {0.5, 0.7, 0.9} | step 0.2 |
| **CFR: λ₁, λ₂ (L2/etc.)** | {1e-3, 1e-4, 1e-5, 1e-6} |  |
| **CNF (Stage-2): λ₁, λ₂** | {1e-3, 1e-4, 1e-5, 1e-6} |  |
| **Learning rate** | {1…9}×{10^−2^,10^−3^,10^−4^} |  |
| **Dropout rate** | {0, 0.10, 0.15, 0.20} | sampled uniformly per call |
| **Bayesian optimization calls** | n_calls = 200 | gp_minimize |
